# Improving cataract services: better access, better outcomes, better value

**Published:** 2022-12-16

**Authors:** David Yorston, Fatima Kyari, John Buchan

**A balanced approach to outcomes, output and outlay – together with strong partnerships – create cataract services that put patients at the centre and deliver better eye health for all.**

**Figure:**
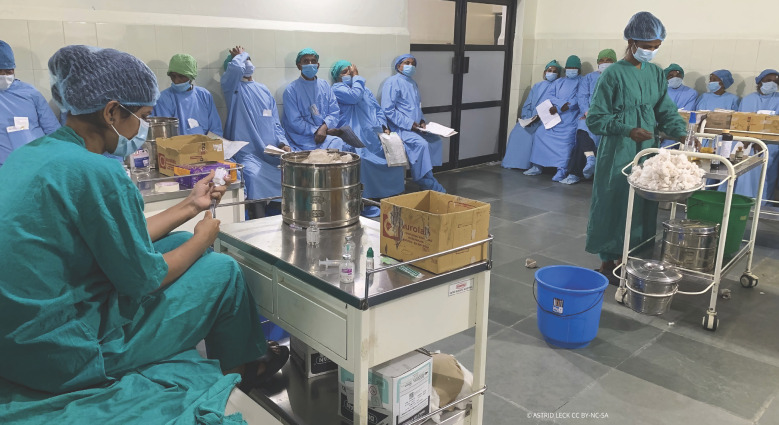
Outcome monitoring and efficient systems allow this hospital to provide high quality cataract surgery to nearly 50,000 patients per year, while costing patients less than US $10 per eye. **NEPAL**

Although highly effective treatment for cataract has been available around the world for several decades, it remains the leading cause of avoidable blindness. It is completely unacceptable that millions of people are deprived of their right to sight due to a condition that can be cured with a safe, fast, and cost-efficient procedure.

The articles in this issue show that improvement doesn’t only rely on new techniques, drugs, or equipment. Instead, improvement is also the result of a coordinated effort by everyone in the eye team to provide a patient-centred service.

The three pillars of combatting vision impairment due to cataract are:

- **Output** – the number of cataract operations performed, often expressed as the cataract surgical rate (the number of cataract operations per million population per year)
- **Outcome** – the results of cataract surgery, i.e., what percentage of eyes achieve good vision after a cataract operation, and the complication rate
- **Outlay** – how much an eye service needs to spend to provide cataract surgery (which will affect how much patients have to pay).

Improving cataract services means addressing all three of these. With a balanced approach to output, outcome, and outlay, it is possible to see major improvements in all three areas.

## How can this be achieved?

The key element – as is evident in the articles in this issue – is partnership. First, partnership with **patients**. More work is needed to understand what matters to patients, so that we can ensure that cataract services are accessible and appropriate to our service users. It is worth considering how you could find out more about what matters to your cataract patients – the answers may surprise you. City Hospital, Nairobi already provides high quality services, but that didn’t stop its leadership from asking patients how they could improve (page 4). The survey highlighted that patients wanted a telephone number to call if they had concerns, and that more patients than expected found the operation painful, prompting a review of their local anaesthesia policy.

The second partnership is with the **community**. There are numerous examples of engagement with the community in this issue of the journal, and in our previous issue on community engagement. These partnerships can involve working together to promote and publicise cataract services. Community partners may be community organisations, local government, businesses, media organisations, faith-based agencies, educational institutions, and patients who are happy with their cataract surgery. Partnerships can involve collaboration in the delivery of services – using a school as a venue for an outreach eye clinic during the weekend, for example. Members of the community can also be trained to identify cataract patients and to support follow-up care after cataract surgery. The greater the involvement of the local community, the more likely it is that patients will know about the services and trust their local eye care provider. Think about your clinic’s links to the local community. Are there avenues of collaboration that you haven’t explored? Are there strong local organisations that could help to promote or deliver cataract services? What about cost sharing models, such as health insurance?

The third partnership is with hospital **management**. In hospitals and eye clinics, in both high- and low-income countries, there needs to be a balance between income generation and cost-containment required by managers, and the scope of service provision by clinicians. This can sometimes lead to conflict: as clinicians, we want to provide the best possible services for everyone who needs them, regardless of the cost; however, managers have a responsibility to balance the books and to ensure that the clinic has enough funds to pay salaries at the end of the month. If we want to treat more patients (increase output), and obtain the best possible results (improve outcomes), we need to acknowledge that this will cost more (increased outlay), and we will need managers to approve the additional expenditure. Fortunately, all parties can achieve their goals. If the number of operations is increased, the unit cost per operation will decrease. This will bring in more profit that can be reinvested in improved services, or in subsidies for patients who would otherwise be unable to afford surgery. Increased outlay is therefore entirely compatible with greater financial sustainability.

The fourth partnership is with eye care **personnel** – the eye team. The most valuable resource an eye clinic has is its workforce, and we need to ensure fair and transparent human resource policies in which all staff members contribute responsibly in their defined roles and are treated fairly and without favouritism. It takes time and effort to build this kind of partnership – one that is based on trust and understanding of the different but complementary needs and objectives of managers, support personnel, and clinicians. Have you ever spoken to the clinic’s receptionist, or the hospital administrator, outside of a formal meeting in the workplace? If not, maybe it is time to start to build these partnerships.

We have a duty to reduce vision impairment caused by cataract, and this issue of the journal offers some pointers. If we keep in mind the essential messages of **partnership**, and balancing **output, outcome,** and **outlay**, then we will be successful.

**Effective Cataract Surgical Coverage (eCSC): improving quality, output and access**

Governments and international organisations, like the World Health Organization (WHO), need to be able to evaluate how well eye health services are doing in reducing avoidable blindness. In the past, they looked just at **quantity**: the number of people in a population who had undergone cataract surgery, using a measurement known as **Cataract Surgical Coverage (CSC)**.

This compared the number of people who had undergone cataract surgery to those who needed surgery (both operated and unoperated), and expressed this as a percentage. CSC did not measure the quality of surgery: how well the patients could see after their cataract operation.

To ensure that **quantity and quality** are both measured, ministries of health, WHO and other institutions increasingly want to know the **Effective Cataract Surgical Coverage (eCSC):** the number of people who can now see well after cataract surgery, expressed as a percentage of those who needed surgery (both operated and unoperated).

In 2021, all WHO Member Countries agreed to a new global target: increasing eCSC by 30 percentage points by 2030.^1,2^ This target sets a new standard for the visual outcome of cataract surgery: a presenting visual acuity (PVA) of 6/12 or better, which is more difficult to achieve than the previous standard: PVA of 6/18 or better.^1^

Increasing eCSC requires that eye units provide high quality surgery – which means routine measurement and reporting of surgical outcomes is now more important than ever. Recording who is coming for surgery is also vital so that we can ensure we are providing equitable access for all, including women and people with disabilities.

Providing people-centred cataract surgery, through outreach services and integration with existing health care services at primary level (as detailed in our recent issues on primary eye health care^3^ and community engagement^4^) will also help to improve patients’ awareness and acceptance of surgery, as well as their ability to physically reach the services they need.

*With thanks to Elmien Wolvaardt, Jacqui Ramke and Heiko Philippin.*

References1KeelSMullerABlockS, et al. Keeping an eye on eye care: monitoring progress towards effective coverage.
Lancet Glob Health.
2021; 9 (10): e1460–4.3423726610.1016/S2214-109X(21)00212-6PMC84402222World Health Organization.
Global eye care targets endorsed by Member States at the 74th World Health Assembly.
**https://bit.ly/eCSC**3Primary eye health care.
Community Eye J.
2021;34 (113). **www.cehjournal.org/primary-eye-care/**PMC9412109360334114Community engagement and patient-centred care.
Community Eye J.
2022;35 (115). **https://bit.ly/CEHJcommunity**
